# Public mobility data enables COVID-19 forecasting and management at local and global scales

**DOI:** 10.1038/s41598-021-92892-8

**Published:** 2021-06-29

**Authors:** Cornelia Ilin, Sébastien Annan-Phan, Xiao Hui Tai, Shikhar Mehra, Solomon Hsiang, Joshua E. Blumenstock

**Affiliations:** 1 School of Information, U.C. Berkeley, Berkeley, USA; 2 Goldman School of Public Policy, U.C. Berkeley, Berkeley, USA; 3 Agricultural and Resource Economics, U.C. Berkeley, Berkeley, USA; 4 National Bureau of Economic Research and Centre for Economic Policy Research, Cambridge, USA

**Keywords:** Diseases, Health care

## Abstract

Policymakers everywhere are working to determine the set of restrictions that will effectively contain the spread of COVID-19 without excessively stifling economic activity. We show that publicly available data on human mobility—collected by Google, Facebook, and other providers—can be used to evaluate the effectiveness of non-pharmaceutical interventions (NPIs) and forecast the spread of COVID-19. This approach uses simple and transparent statistical models to estimate the effect of NPIs on mobility, and basic machine learning methods to generate 10-day forecasts of COVID-19 cases. An advantage of the approach is that it involves minimal assumptions about disease dynamics, and requires only publicly-available data. We evaluate this approach using local and regional data from China, France, Italy, South Korea, and the United States, as well as national data from 80 countries around the world. We find that NPIs are associated with significant reductions in human mobility, and that changes in mobility can be used to forecast COVID-19 infections.

## Introduction

Societies and decision-makers around the globe are deploying unprecedented non-pharmaceutical interventions (NPIs) to manage the COVID-19 pandemic. These NPIs have been shown to slow the spread of COVID-19^1–4^, but they also create enormous economic and social costs (for example^5–10^). Thus, different populations have adopted wildly different containment strategies^11^, and local decision-makers face difficult decisions about when to impose or lift specific interventions in their community. In some contexts, these decision-makers have access to state-of-the-art models, which simulate potential scenarios based on detailed epidemiological models and rich sources of data (for example^12,13^).

In contrast, many local and regional decision-makers do not have access to state-of-the-art epidemiological models, but must nonetheless manage the COVID-19 crisis with the resources available to them. With global public health capacity stretched thin by the pandemic, thousands of cities, counties, and provinces—as well as many countries—lack the data and expertise required to develop, calibrate, and deploy the sophisticated epidemiological models that have guided decision-making in regions with greater modeling capacity^14–16^. In addition, early evidence suggests a need to adapt models to a local context, particularly for developing countries, where disease, population and other characteristics are different from developed countries, where models are primarily being developed^17–19^.

Here, we aim to address this “modeling-capacity gap” by developing, demonstrating, and testing a simple approach to forecasting the impact of NPIs on infections. This approach is built on two main insights. First, we show that passively collected data on human mobility, which has previously been used to measure NPI compliance^20–26^, can also effectively forecast the COVID-19 infection response to NPIs up to 10 days in the future. Second, we show that basic concepts from econometrics and machine learning can be used to construct these 10-day forecasts, effectively emulating the behavior of more sophisticated epidemiological models, including those which incorporate mobility data^27,28^.

This approach is not a substitute for more refined epidemiological models. Rather, it represents a practical and low-cost alternative that may be easily adopted in many contexts when the former is unavailable. It is designed to enable any individual with access to standard statistical software to produce forecasts of NPI impacts with a level of fidelity that is practical for decision-making in an ongoing crisis.

## Data

**Figure 1:**
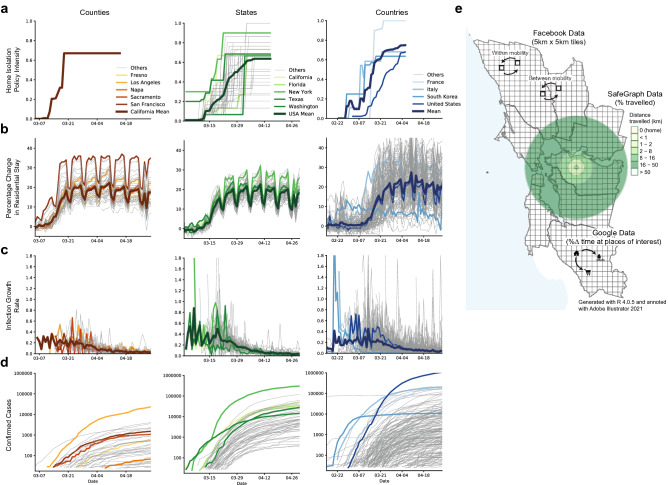
Data on mobility measures, COVID-19 infections and home isolation policy adoption. (**a**) Home isolation policy adoption, (**b**) Change in time spent at home, (**c**) Infection growth rate, and (**d**) Total confirmed cases are displayed at the county, state and country level. (**e**) Illustrative example of different mobility measures in California. We utilize data on trips both within and between counties (Facebook and Baidu) as well as the purpose of the trip (Google) and the average distance traveled (SafeGraph).

Our study links information on non-pharmaceutical interventions (NPIs, shown in Fig. 1a) to patterns of human mobility (Fig. 1b) and COVID-19 cases (Figure 1c-d). All data were obtained from publicly available sources. We provide a brief summary of these data here; full details are provided in “Supplementary file 1: Appendix A”.

**Figure 2:**
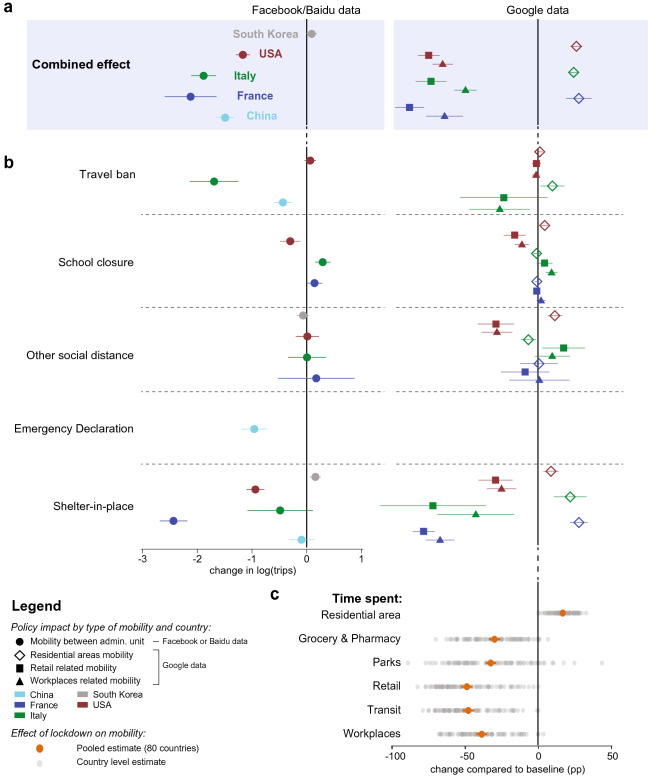
Empirical estimates of the effect of NPIs on mobility measures. Markers are country specific-estimates, whiskers show the 95% confidence interval. (**a**) Estimated combined effect of all policies on number of trips between counties (left) and time spent in specific places (right). (**b**) Estimated effects of individual policy or policy groups on mobility measures, jointly estimated for each country. (**c**) Estimated effect of lockdown on mobility the 80 countries which experienced such policy, jointly estimated for each type of mobility.

### Non-pharmaceutical interventions

We obtain NPI data from two sources. At the sub-national level, we use the NPI dataset compiled by Global Policy Lab^2,29^. For each sub-national region in five countries, we observe the fraction of the population treated with NPIs in each location on each day. We aggregate 13 different policy actions into four general categories: Shelter in Place, Social Distance, School Closure, and Travel Ban. At the national level, we compiled data on national lockdown policies from the Organisation for Economic Co-operation and Development (OECD)—Country Policy Tracker^30^, and crowed-sourced information on Wikipedia and COVID-19 Kaggle competitions^31^.

### Human mobility

We source publicly-available data on human mobility from Google, Facebook, Baidu and SafeGraph. These private companies provide free aggregated and anonymized information on the movement of users of their online platform (Fig. 1e). Data from Google indicates the percentage change in the amount of time people spend in different types of locations (e.g., residential, retail, and workplace)^32^. These changes are relative to a baseline defined as the median value, for the corresponding day of the week, during Jan 3–Feb 6, 2020. Facebook provides estimates of the number of trips within and between square tiles (of resolution up to 360m$^2$) in a region^33^. We aggregate these data to show trips between and within sub-national units. Baidu provides similar data, indicating movement between and within major Chinese cities^34,35^. Lastly, SafeGraph dataset gives us information on average distance travelled from home by millions of devices across the US^36^.

### COVID-19 cases

For each sub-national and national unit, we obtain the cumulative confirmed cases of COVID-19 from the data repository compiled by the Johns Hopkins Center for Systems Science and Engineering (CSSE)^37^. The World Health Organization (WHO) provides similar data at the national level but at the moment of writing this paper, no such data are available at the sub-national level^38^.

### Linking data sets

The availability of epidemiological, policy, and mobility data varies across subnational units and countries included in the analysis. We distinguish between three different levels of aggregation for administrative regions - denoted “ADM2” (the smallest unit), “ADM1”, “ADM0.” Our global analysis is conducted using ADM0 data. The country-specific analysis is determined by data availability. Results are provided at the prefecture (ADM2) and province level (ADM1) in China; the regional (ADM1) level in France; the province (ADM2) and region (ADM1) level in Italy; the province (ADM1) level in South Korea; and the county (ADM2) and state (ADM1) level in the United States.

We merge the sub-national NPI, mobility, and epidemiological data based on administrative unit and day to form a single longitudinal (panel) data set for each country. We merge the daily country-level observations to construct a longitudinal data sets for the portion of the world we observe.

## Methods

We briefly summarize our methodology below. This discussion is meant to be accessible to a general audience, including policymakers who do not necessarily have advanced training in statistics. Full details, including model equations and estimation methods, are provided in “Supplementary file 1: Appendix B”.

### Models

We decompose the impact of an NPI on infections ($\frac{\Delta infections}{\Delta NPI}$) into two components that can be modeled separately: the change in behavior associated with the NPI, and the resulting change in infections associated with that change in behavior:

1 $$\begin{aligned} \frac{\Delta infections}{\Delta NPI} = \frac{\Delta behavior}{\Delta NPI} \times \frac{\Delta infections}{\Delta behavior}. \end{aligned}$$

We construct models to describe each of these two factors. The “behavior model” describes how mobility behavior changes in association with the deployment of NPIs ($\frac{\Delta behavior}{\Delta NPI}$). The “infection model” describes how infections change in association with changes in mobility behavior ($\frac{\Delta infections}{\Delta behavior}$). Both models are “reduced-form” models, commonly used in econometrics, that characterize the behavior of these variables without explicitly modeling the underlying mechanisms that link them (cf.^2^). Instead, these models emulate the output one would expect from more sophisticated and mechanistically explicit epidemiological models—without requiring the underlying processes to be specified. While this reduced-form approach does not provide the same epidemiological insight that more detailed models do, they demand less data and fewer assumptions. For example, they can be fit to local data by analysts with basic statistical training, not necessarily in epidemiology, and they do not require knowledge of fundamental epidemiological parameters—some of which may differ in each context and can be difficult to determine. The performance of these simple, low-cost models can then be evaluated via cross-validation, i.e., by systematically evaluating out-of-sample forecast quality.

#### Behavior model

For each country, we separately estimate how daily sub-national mobility behavior changes in association with the deployments of NPIs using a country-specific model. In the global model, we pool data across countries and estimate how mobility in each country changes in association with national exposure to NPIs. Each category of mobility on each day is assumed to be simultaneously influenced by the collection of NPIs that are active in that location on that day. A panel multiple linear regression model is used to estimate the relative association of each category of mobility with each NPI. Our approach accounts for constant differences in baseline mobility between and within each sub-national unit—such as differences due to regional commuting patterns, culture, or geography, and differences in mobility across days of the week. These effects are not modeled explicitly but instead are accounted for non-parametrically. “Supplementary file 1: Appendix B.1” contains details of the modeling approach.

#### Infection model

As with the behavior model, we model the daily growth rate of infections at the local, national, and global scale. In each location, we model the daily growth rate of infections as a function of recent human mobility and historical infections. The approach does not require epidemiological parameters, such as the incubation period or $R_0$, nor information on NPIs.

In practice, we estimate a distributed-lag model where the predictor variables are mobility rates in that location for the prior 21 days, and the dependent variable is the daily infection growth rate, constructed as the first-difference of log confirmed infections. This approach captures the intuition that human mobility is a key factor in determining rates of infection, but does not require parametric assumptions about the nature of that dependency. The model also accounts for constant differences in baseline infection growth rates within each locality—such as those due to differences in local behavior unrelated to mobility, differences across days of the week, and changes in how confirmed infections are defined or tested for. This approach is also robust to incomplete rates of COVID-19 testing, uneven patterns of testing across space, and gradual changes in testing over time^2^—see “Supplementary file 1: Appendix B.2” for details.

We fit the model using historical data from each location, and follow stringent practices of cross-validation to ensure that the models are not ‘overfit’ to historical trends. The accuracy of the forecast is then evaluated against actual infections observed during the forecast period, but which were not used to fit the model. Models are fit at the finest administrative level where data are available and forecasts are aggregated to larger regions to evaluate the ability of the model to predict infections at different spatial scales. “Supplementary file 1: Appendix B.2” contains details of the modeling approach.

In principle, such future forecasts can be used by decision-makers who are able to influence local mobility through policy and/or NPIs, perhaps informed either by a behavioral model or observation. Here, we test the quality of the infection model to generate forecasts by simulating and evaluating what a forecaster would have predicted had they generated a forecast at a historical date. In the forecasts presented here, we assume that mobility remains at the level observed during the forecast period—although in practice we expect that decision-makers would simulate different forecasts under different mobility assumptions to inform NPI deployment and policy-making.

## Results

We first present results from our behavior model, characterizing the mobility response of different populations to different NPIs. We then evaluate the infection model’s ability to forecast COVID-19 infections based on these same mobility measures. We conclude by discussing how these models could be used to guide policy decisions at local and regional scales.

### Mobility response to NPIs

We estimate the reduction in human mobility associated with the deployment of NPIs by linking comprehensive data on policy interventions to mobility data from several different countries at multiple geographic scales. We find that the combined impact of all NPIs reduced mobility between administrative units (Facebook/Baidu) by 73% on average across the countries with sub-national policy data (Fig. 2a). The combined effects were of similar magnitude in China (− 78%, se = 8%), France (− 88%, se = 27%), Italy (− 85%, se = 12%), and the US (− 69%, se = 6%); no significant change was observed in South Korea, where mobility was not a direct target of NPIs (for example^39^). Excluding South Korea, we estimate that all policies combined were associated with a decrease in mobility by 81% . The general consistency of these magnitudes across countries holds for alternative measures of mobility: using Google data we find that all NPIs combined result in an increase in time spent at home by 28% (se = 2.9), 24% (se = 1.3), and 26% (se = 1.3) in France, Italy, and the US, respectively. This was achieved, in part, by reducing time spent at workplaces by an average of 59.8% and time in commercial retail locations by an average of 78.8%.

**Figure 3:**
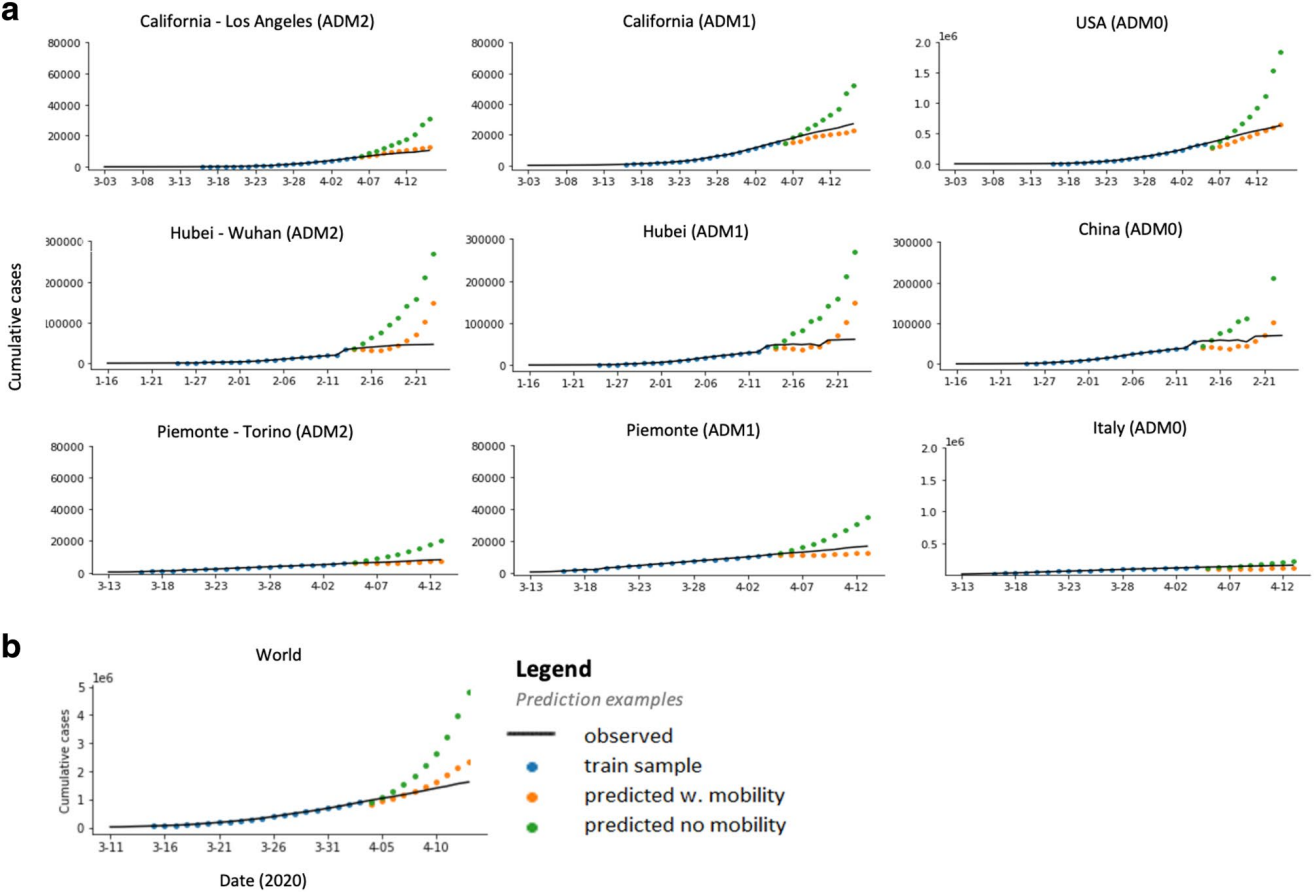
Short term prediction of COVID-19 cases. Solid line is the recorded number of COVID-19 infections, markers show data in our training sample (blue) and our predictions estimated using mobility measures (orange) versus a model without mobility (green). Model with no mobility measures consistently over-predict the number of infections and drift away quickly from the observed data. (**a**) This pattern is confirmed when aggregating locally estimated predictions (left) at the state (middle) and country (right) level. (**b**) Similarly, predictions obtained from country level estimates are significantly more accurate when a measure of mobility is included.

**Figure 4:**
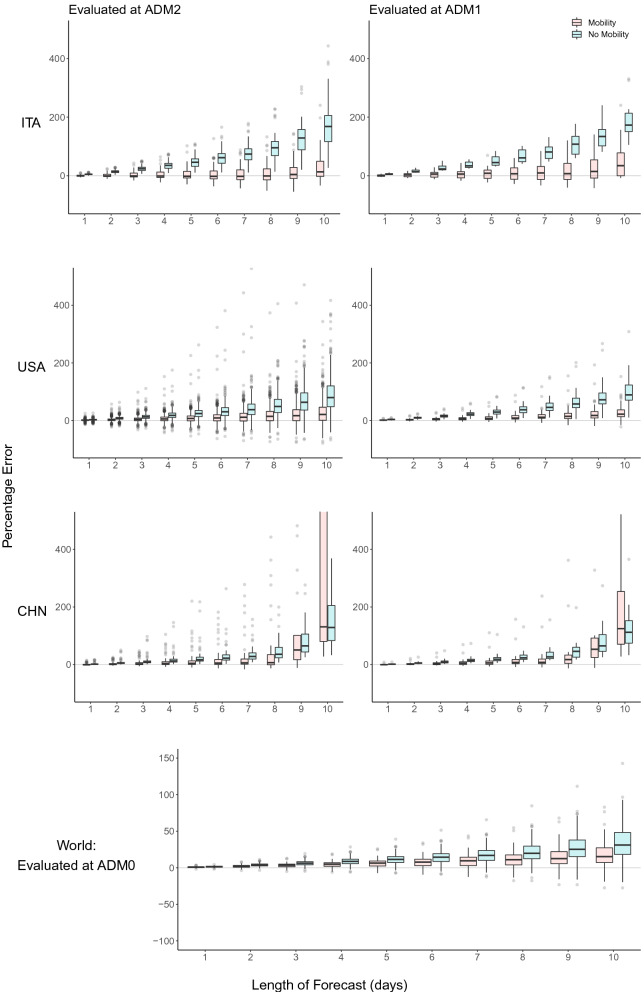
Evaluation of forecast errors for the infection model. For Italy, US and China, forecasts are evaluated at the finest administrative level (ADM2), as well as aggregated to larger regions (ADM1). For each ADM2 region and forecast length, the mean is taken over all available forecast dates, and the error is evaluated using that mean. Boxplots display the distribution of these percentage errors for each ADM2 region. These are then aggregated to ADM1 level (right panel), for both models including and excluding mobility variables. Similarly, for data fitted at a global level (bottom-most plot), for each country and forecast length, the mean is taken over all forecast dates.

We estimate the impact of each individual NPI on total trips (Facebook/Baidu) and quantity of time spent at home and other locations (Google) accounting for the estimated impact of all other NPIs. Travel bans are significantly associated with large mobility reductions in China (− 70%, se = 7%) and Italy (− 82%, se = 25%), where individuals stayed home for 10% more time, but not in the US (Fig. 2b). School closures were associated with moderate negative impacts on mobility in the US (− 26%, se = 10%) and increased time at home (4.6%, se = 0.7%) but slight positive impacts in Italy (33%, se = 7%) and France (15%, se = 7%). Other social distancing policies, such as religious closures, had no consistent impact on total trips but were associated with individuals spending more time at home in the US (11.5%, se = 1.6%) and more time in retail locations in Italy (17.6%, se = 4.8%). Similarly, the national emergency declaration was associated with significant mobility reductions in China (- 62.6 %, se = 12.7 %). Shelter-in-place orders were associated with large reductions in trips for the US (− 60.8%, se = 8%), Italy (− 38.4%, se = 35%), and France (− 91.2%, se = 13.6%), and large increases in the fraction of time spent in homes (8.9%, 22.1%, 28%, respectively). Shelter in place orders did not appear to have large impacts in South Korea or China. This is consistent with earlier policies (such as the Emergency Declaration) restricting movement in China earlier than the shelter in place orders, while mobility in South Korea was never substantially affected by NPIs.

Globally, we find evidence that lockdown policies were associated with substantial reductions in mobility (Fig. 2c). Across 80 countries, the average time spend in non-residential locations decreased by 40% (se = 2%) in response to NPIs. Time spent in retail locations is the most impacted category, declining 49.9% (se = 2%). Some of the variation in response across countries (grey dots) likely reflects different social, cultural, and economic norms; measurement error; and statistical variability. In “Supplementary file 1: Appendix C”, we disaggregate this effect temporally, and find that the most significant reductions occur during the first eight days after a lockdown (Figure S1c).

In “Supplementary file 1: Appendix C”, we further exploit the granular resolution of the mobility data to investigate whether localized policies also impacted neighboring regions (Figure S1). In the USA and Italy, the impact of NPIs on mobility was highly localized, with little evidence of spatial spillover effects (“Supplementary file 1: Appendix C - Figure S1a”). In China, the evidence is more mixed, with some evidence of spillovers between neighboring cities (“Supplementary file 1: Appendix C - Fig S1b”).

### Forecasting infections based on mobility

We find that mobility data alone are sufficient to meaningfully forecast COVID-19 infections 7–10 days ahead at all geographic scales – from counties and cities (ADM2), to states and provinces (ADM1), to countries (ADM0) and the entire world. Furthermore, identical models that exclude mobility data perform substantially worse, suggesting an important role for mobility data in forecasting.

Figure 3 illustrates the performance of model forecasts in several geographic regions and at multiple scales. The true infection rate is shown as a solid line; data used to train each model are depicted in blue dots, and the forecast of our model is shown in orange, contrasted against a model with no mobility data in green. Forecasts that account for current and lagged measures of mobility generally track actual cases more closely than forecasts that do not account for mobility. For example, a forecast made for the period 4/06/2020–4/15/2020 for California-Los Angeles on 4/15/2020 without mobility projects 30,716 cases, while the same forecast accounting for mobility would be 12,650 cases, much closer to the 10,496 that was observed. Figure 3b depicts projected cases for the entire world based on this reduced-form approach, estimated using country-level data mobility data from Google.

Figure 4 summarizes model performance across *all* administrative subdivisions of each of the three countries we consider for the forecast analysis (China, Italy, and the United States). We show the distribution of model errors over all ADM2 and ADM1 regions at forecast lengths ranging from 1 to 10 days. Table 1 summarizes each distribution using the median.

**Ta﻿ble 1:** Median percentage error for each model and day of forecast, as plotted in Fig. 4. The error is presented for each model and geographical region, and for 1 to 10 day forecasts.

Model	Country	Level	Median percentage error (MPE) for forecast length (days)									
			1	2	3	4	5	6	7	8	9	10
Mobility	World	ADM0	0.90	2.19	3.46	4.83	6.35	7.80	9.54	11.00	12.60	15.24
No Mobility	World	ADM0	1.50	3.84	6.32	8.70	11.46	14.42	16.91	19.75	25.28	31.12
Mobility	China	ADM1	−0.20	1.41	3.05	4.55	5.91	7.54	7.60	17.59	53.26	124.82
No Mobility	China	ADM1	1.47	5.39	8.84	14.10	18.45	23.45	26.80	45.86	64.95	112.03
Mobility	China	ADM2	−0.44	0.89	2.11	3.29	4.18	5.19	6.10	6.80	50.66	131.09
No Mobility	China	ADM2	1.37	5.17	8.59	12.48	16.83	22.33	28.14	35.70	65.09	128.80
Mobility	Italy	ADM1	0.89	2.70	5.60	5.68	8.29	6.65	9.31	6.83	14.39	34.19
No Mobility	Italy	ADM1	4.86	13.66	23.12	33.69	45.03	61.06	80.95	107.48	133.98	172.74
Mobility	Italy	ADM2	−0.87	−0.90	−0.81	−1.06	−1.73	−2.11	−2.08	−0.99	4.41	13.27
No Mobility	Italy	ADM2	4.96	14.20	23.75	35.26	45.81	61.66	73.96	95.47	128.89	167.97
Mobility	US	ADM1	1.13	2.62	3.97	5.45	6.68	8.52	11.19	14.28	17.24	21.82
No Mobility	US	ADM1	3.50	9.39	15.29	21.79	29.22	37.11	45.54	57.36	71.72	88.73
Mobility	US	ADM2	0.98	2.30	3.64	5.21	7.00	8.77	11.00	14.28	16.67	20.75
No Mobility	US	ADM2	2.80	7.72	12.62	17.79	23.79	30.34	37.37	48.43	63.31	79.47

In all geographies and at all scales, models with mobility data perform better than models without. In general, sub-national forecasts in China benefit least from mobility data, but forecasts in Italy and the US are substantially improved by including a single measure of mobility for the 21 days prior to the date of the forecast. At the local (ADM2) level in Italy, the MPE is −1.73% and 13.27% for five and ten days in the future when mobility is accounted for, compared to 45.81% and 167.97% when it is omitted. In the US, MPE is 7.00% (5-day) and 20.75% (10-day) accounting for mobility, and 23.79% and 79.47% omitting mobility. In China, MPE is 4.18% (5-day) and 131.09% (10-day) accounting for mobility, and 16.83% and 128.80% omitting mobility. At the regional (ADM1) level, MPE rates are similar but extreme errors are reduced, largely because positive and negative errors cancel out. Country-level forecasts, which use country-level mobility data from Google, benefit relatively less than sub-national model from including mobility information, in part because baseline forecast errors are smaller. For countries in our sample, MPE is 6.35% (5-day) and 15.24% (10-day) accounting for mobility, and 11.46% and 31.12% omitting mobility.

### Model application in decentralized management of infections

Our results suggest that a simple reduced-form approach to estimating model (1) may provide useful information and feedback to decision-makers who might otherwise lack the resources to access more sophisticated scenario analysis. We imagine the approach can be utilized in two ways. First, a decision-maker considering an NPI (either deploying, continuing, or lifting) could develop an estimate for how that NPI might affect behavior, based on our analysis of different policies above (Fig. 2). Using these estimated changes in mobility, they could then forecast changes in infections using the infection model described above—but fit to local data.

Table 2 provides an example calculation for how a novel policy that increased residential time (observed in Google data) would alter future infections, using estimates from the global-level model. For example, a policy that increases residential time by 5% in a country is predicted to reduce cumulative infections ten days later, to 82.5% (CI: (78.2, 87.0)) of what they would otherwise have been. Similar tabulations can be generated by fitting infection models using recent and local data, which would flexibly capture local social, economic, and epidemiological conditions.

**Table 2:** Example: Estimating the effect of a mobility-reducing policy on infections for the global model (unit of observation is a country day). The values in the table are the ratio of the cumulative number of cases after up to 10 days, if residential time over baseline was increased in a country *at day 0* by $\Delta$ = 1%, 5%, 10% or 20% from their original values. These values are estimated using coefficients of the mobility variables derived from the pooled global model (details in “Supplementary file 1: Appendix B.2”). Note that each column compares to the value on its first row (indicated by the value 1). An example interpretation is: if a country increases residential time by 5%, cumulative infections ten days later is predicted to be 82.5% of what they would have been with no change in mobility.

$\Delta$	Day = 1	2	3	4	5	6	7	8	9	10
0	1	1	1	1	1	1	1	1	1	1
.01	1	0.998	0.996	0.993	0.989	0.985	0.98	0.975	0.969	0.962
.05	0.998	0.992	0.981	0.966	0.948	0.928	0.906	0.881	0.854	0.825
.10	0.996	0.983	0.962	0.934	0.899	0.862	0.821	0.776	0.729	0.68
.20	0.992	0.967	0.926	0.873	0.808	0.743	0.674	0.603	0.532	0.463

A second way that a decision-maker could use our approach would be to actually deploy a policy without *ex ante* knowledge of the effect it will have on mobility, instead simply observing mobility responses that occur after NPI deployment using these publicly available data sources. Based on these observed responses, they could forecast infections using our behavior model.

## Discussion

The COVID-19 pandemic has led to an unprecedented degree of cooperation and transparency within the scientific community, with important new insights rapidly disseminated freely around the globe^40^. However, the capacity of different populations to leverage new scientific insights is not uniform. In many resource-constrained contexts, critical decisions are not supported by robust epidemiological modeling of scenarios. Here we have demonstrated that freely available mobility data can be used in simple models to generate practically useful forecasts. The goal is for these models to be accessible to a single individual with basic training in regression analysis using standard statistical software. The reduced-form model we develop generally performs well when fit to local data, except in China where it cannot account for some key factors that contributed to reductions in transmission.

A key insight from our work is that passively observed measures of aggregate mobility are useful predictors of growth in COVID-19 cases. However, this does not imply that population mobility itself is the only fundamental cause of transmission. The measures of mobility we observe capture a degree of “mixing” that is occurring within a population, as populations move about their local geographic context. This movement is likely correlated with other behaviors and factors that contribute to the spread of the virus, such as low rates of mask-wearing and/or physical distancing. Our approach does not explicitly capture these other factors—and thus should not be used to draw causal inferences—but is possible that our infection model performs well in part because the easy-to-observe mobility measures capture these other factors by proxy.

The simple model we present here is designed to provide useful information in contexts when more sophisticated process-based models are unavailable, but it should not necessarily displace those models where they are available. In cases where complete process-based epidemiological models have been developed for a population and can be deployed for decision-making, the model we develop here could be considered complementary to those models. Future work might determine how information from combinations of qualitatively distinct models can be used to optimally guide decision-making.

We also note that the reduced-form model is designed to forecast infections in a certain population at a restricted point in time. It achieves this by capturing dynamics that are governed by many underlying processes that are unobserved by the modeler. However, because these underlying mechanisms are only captured implicitly, the model is not well-suited to environments where these underlying dynamics change dramatically. In such circumstances, process-based models will likely perform better. The reduced-form approach presented here can still be applied in such circumstances, but it may be necessary to refit the model based on data that is representative of current conditions. Similarly, when our reduced-form model is applied to a new population, it should be fit to local data to capture dynamics representative of the new population.

The approach we present here depends critically on the availability of aggregate mobility data, which is currently provided to the public by private firms that passively collect this information. At the time of writing, these mobility datasets are publicly available in 135 and 152 countries for Google and Facebook, respectively. In lower-resource settings, where use of smartphones is less common, the users who generate mobility data may not be as representative of the total population as in wealthy nations, but prior work suggests that biases in phone ownership may not dramatically bias estimates of overall population mobility^41^. In such contexts, anonymized metadata from mobile phone operators is increasingly being made available for research and policy interventions^42,43^, and offers a promising source of data for public health applications^44^.

We hypothesize that the approach we develop here might skillfully forecast the spread of other diseases besides COVID-19. If true, this suggests our approach could provide useful information to decision-makers for managing other public health challenges, such as influenza or other outbreaks, potentially indicating a public health benefit from firms continuing to made mobility data available—even after the COVID-19 pandemic has subsided.

## Supplementary information

Supplementary material 1 (pdf 765 KB)

## Data Availability

Data used in this study can be divided into three categories - Epidemiological, Policy and Mobility. They are publicly available at different locations. We collected epidemiological data from the 2019 Novel Coronavirus COVID-19 (2019-nCoV) Data Repository compiled by the Johns Hopkins Center for Systems Science and Engineering (JHU CSSE)^37^. The policy data was constructed and made available for academic research by Global Policy Lab^2,29^. Mobility data comes from three of the biggest internet companies - Google, Facebook and Baidu. Google mobility data summarizes time spent by their users each day after Feb 6, 2020 in various types of places, such as residential, workplaces and grocery stores^32^. Facebook summarizes and anonymizes its user data into useful metrics that can be used to evaluate the movement of people^33^. Baidu provides aggregated user location data and mobility metrics via its Smart Eye Platform^36^. A dump of all datasets analysed during the study are also available from the corresponding author on reasonable request.

## References

[1] Chinazzi M (2020). The effect of travel restrictions on the spread of the 2019 novel coronavirus (covid-19) outbreak. Science.

[2] Hsiang, S. *et al.* The effect of large-scale anti-contagion policies on the covid-19 pandemic. *Nature* 1–9 (2020). http://www.globalpolicy.science/covid19. 10.1038/s41586-020-2404-8 32512578

[3] Ferguson, N. *et al.* Report 9: impact of non-pharmaceutical interventions (npis) to reduce covid19 mortality and healthcare demand (2020). 10.1007/s11538-020-00726-x PMC7140590 32270376

[4] Tian H (2020). An investigation of transmission control measures during the first 50 days of the covid-19 epidemic in china. Science.

[5] Gössling S, Scott D, Hall CM (2020). Pandemics, tourism and global change: a rapid assessment of covid-19. J. Sustain. Tour..

[6] Atkeson, A. *What Will be the Economic Impact of Covid-19 in the Us? Rough Estimates of Disease Scenarios*. Technical Report, National Bureau of Economic Research (2020).

[7] Coibion, O., Gorodnichenko, Y. & Weber, M. *The Cost of the Covid-19 Crisis: Lockdowns, Macroeconomic Expectations, and Consumer Spending*, Technical Report, National Bureau of Economic Research (2020).

[8] Thunström L, Newbold SC, Finnoff D, Ashworth M, Shogren JF (2020). The benefits and costs of using social distancing to flatten the curve for covid-19. J. Benefit Cost Anal..

[9] Rossi R (2020). Covid-19 pandemic and lockdown measures impact on mental health among the general population in Italy. Front. Psychiatry.

[10] Zhang, S. X. *et al.* Succumbing to the covid-19 pandemic-healthcare workers not satisfied and intend to leave their jobs. *Int. J. Mental Health Addict.* **1–10** (2021). 10.1007/s11469-020-00418-6 PMC7790354 33437225

[11] Cheng C, Barceló J, Hartnett AS, Kubinec R, Messerschmidt L (2020). Covid-19 government response event dataset (coronanet v. 1.0). Nat. Hum. Behav..

[12] Friedman, J., Liu, P., Gakidou, E., COVID, I. & Team, M. C. Predictive performance of international covid-19 mortality forecasting models. *medRxiv* (2020). 10.1038/s41467-021-22457-w PMC8110547 33972512

[13] Ray, E. L. *et al.* Ensemble forecasts of coronavirus disease 2019 (covid-19) in the us. *medRxiv* (2020).

[14] Liverani, M., Hawkins, B. & Parkhurst, J. O. Political and institutional influences on the use of evidence in public health policy. a systematic review. *PloS one* **8**, e77404 (2013). 10.1371/journal.pone.0077404 PMC3813708 24204823

[15] Gnanvi, J. E., Kotanmi, B. *et al.* On the reliability of predictions on covid-19 dynamics: a systematic and critical review of modelling techniques. *medRxiv* (2020). 10.1016/j.idm.2020.12.008 PMC7802527 33458453

[16] Loembé, M. M. *et al.* Covid-19 in africa: the spread and response. *Nat. Med.* **1–4** (2020). 10.1038/s41591-020-0961-x 32528154

[17] Twahirwa Rwema, J. O. *et al.* Covid-19 across Africa: epidemiologic heterogeneity and necessity of contextually relevant transmission models and intervention strategies (2020). 10.7326/M20-2628 PMC7384264 32551812

[18] Evans MV (2020). Reconciling model predictions with low reported cases of covid-19 in sub-Saharan Africa: Insights from Madagascar. Global Health Action.

[19] Mueller, V., Sheriff, G., Keeler, C. & Jehn, M. Covid-19 policy modeling in sub-Saharan Africa. *Appl. Econ. Perspect. Policy* (2020).

[20] Engle, S., Stromme, J. & Zhou, A. Staying at home: mobility effects of covid-19. Available at SSRN (2020).

[21] Morita, H., Kato, H. & Hayashi, Y. International comparison of behavior changes with social distancing policies in response to covid-19. Available at SSRN 3594035 (2020).

[22] Wellenius, G. A. *et al.* Impacts of state-level policies on social distancing in the united states using aggregated mobility data during the covid-19 pandemic. arXiv preprint arXiv:2004.10172 (2020).

[23] Pepe, E. *et al.* Covid-19 outbreak response: a first assessment of mobility changes in italy following national lockdown. *medRxiv* (2020). 10.1038/s41597-020-00575-2 PMC7343837 32641758

[24] Klein, B. *et al.* Assessing changes in commuting and individual mobility in major metropolitan areas in the united states during the covid-19 outbreak (2020).

[25] Kraemer MUG (2020). The effect of human mobility and control measures on the COVID-19 epidemic in China. Science.

[26] Martín-Calvo, D., Aleta, A., Pentland, A., Moreno, Y. & Moro, E. Effectiveness of social distancing strategies for protecting a community from a pandemic with a data driven contact network based on census and real-world mobility data. In *Technical Report* (2020).

[27] Malani, A. *et al.**Adaptive control of covid-19 outbreaks in india: local, gradual, and trigger-based exit paths from lockdown*. Technical Report, National Bureau of Economic Research (2020).

[28] Chang, S. *et al.* Mobility network models of covid-19 explain inequities and inform reopening. *Nature* **1–6** (2020). 10.1038/s41586-020-2923-3 33171481

[29] Global Policy Lab. *UC Berkeley* (2020). http://www.globalpolicy.science/covid19.

[30] *The Organisation for Economic Co-operation and Development* (2020). https://www.oecd.org/coronavirus/en/#country-tracker.

[31] COVID-19 lockdown dates by country. *Kaggle* (2020). https://www.kaggle.com/jcyzag/covid19-lockdown-dates-by-country.

[32] COVID-19 Community Mobility Reports. *Google* (2020). https://www.google.com/covid19/mobility/.

[33] Facebook Disaster Maps. *Facebook* (2020). research.fb.com/publications/facebook-disaster-maps-aggregate-insights-for-crisis-response-recovery.

[34] Spatio-temporal Big Data Service. *Baidu* (2020). https://huiyan.baidu.com.

[35] China-Data-Lab. Baidu Mobility Data. *Harvard Dataverse* (2020). 10.7910/DVN/FAEZIO.

[36] Social Distancing Metrics. *SafeGraph* (2020). https://docs.safegraph.com/docs/social-distancing-metrics.

[37] COVID-19 Data Repository by the Center for Systems Science and Engineering (CSSE). *Johns Hopkins University* (2020). https://github.com/CSSEGISandData/COVID-19.

[38] COVID-19 Data Repository by the World Health Organization. *World Health Organization* (2020). https://covid19.who.int.

[39] You J (2020). Lessons from South Koreas covid-19 policy response. Am. Rev. Public Admin..

[40] Zastrow M (2020). Open science takes on the coronavirus pandemic. Nature.

[41] Wesolowski A, Eagle N, Noor AM, Snow RW, Buckee CO (2013). The impact of biases in mobile phone ownership on estimates of human mobility. J. R. Soc. Interface.

[42] Blumenstock, J. Machine learning can help get covid-19 aid to those who need it most. *Nature (Lond.)* (2020). 10.1038/d41586-020-01393-7 32409767

[43] Blondel, V. D. *et al.* Data for development: the d4d challenge on mobile phone data. arXiv preprint arXiv:1210.0137 (2012).

[44] Oliver, N. *et al.* Mobile phone data for informing public health actions across the covid-19 pandemic life cycle (2020). 10.1126/sciadv.abc0764 PMC7274807 32548274

