# Supplementary material for: Public mobility data enables COVID-19 forecasting and management at local and global scales
Source: Sci Rep. 2021 Jun 29;11:13531. doi: 10.1038/s41598-021-92892-8 (PMC8241991; doi:10.1038/s41598-021-92892-8)
Supplement: Supplementary file 1 — Supplementary material 1 (pdf 765 KB) [file 41598_2021_92892_MOESM1_ESM.pdf]

Appendices

A Data Acquisition and Processing

Data used in this study can be divided into three categories - Epidemiological, Policy and Mobility. The sources of these data sets include various research institutions, government public health websites, regional newspaper articles and digital social media platforms.

A.1 Epidemiological Data

We collected epidemiological data from the 2019 Novel Coronavirus COVID-19 (2019-nCoV) Data Repository compiled by the Johns Hopkins Center for Systems Science and Engineering (JHU CSSE).[36] The primary variable of interest for our study is *cum_confirmed_cases*, i.e., the total number of confirmed positive cases in an administrative area since the first confirmed case. We accessed it along with other relevant metadata, including:

date: The date of observation

adm0_name: The ISO3 region (Administrative Level 0) code of the observation

adm1_name: The name of the "Administrative Level 1" region of the observation

adm2_name: The name of the "Administrative Level 2" region of the observation

A.2 Policy data

The policy data was constructed and made available for academic research by Global Policy Lab.[2,29] For each country, the relevant country-specific policies were identified and mapped to four harmonized policy categories - Travel Ban, School Closure, Shelter in place, and Social Distance. These category variables were created by taking an average of policy variables related to that category.

i. Travel Ban

- *travel_ban_local*: Represents a policy that restricts people from entering or exiting the administrative area (e.g., county or province) treated by the policy.

ii. School Closure

- *school_closure*: Represents a policy that closes school and other educational services in that area.

iii. Shelter In Place

- *home_isolation*: Represents a policy that prohibits people from leaving their home regardless of their testing status. For some countries, the policy can also include the case when people have to stay at home, but are allowed to leave for work- or health-related purposes. For the latter case, when the policy is moderate, this is coded as *home_isolation = 0.5*.
- *work_from_home* : Represents a policy that requires people to work remotely. This policy may also include encouraging workers to take holiday/paid time off.
- *business_closure* : Represents a policy that closes all offices, non-essential businesses, and non-essential commercial activities in that area.
- *pos_cases_quarantine* : A policy that mandates that people who have tested positive for COVID-19, or subject to quarantine measures, have to confine themselves at home. The policy can also include encouraging people who have fevers or respiratory symptoms to stay at home, regardless of whether they tested positive or not.
- *welfare_service_closure*: A policy that mandates closure of welfare services such as day care centers for children.
- *emergency_declaration*: Represents a decision made at the city / municipality, county, state / provincial, or federal level to declare a state of emergency. This allows the affected area to marshal emergency funds and resources as well as activate emergency legislation.

iv. Social Distance

- *social_distance*: Represents a policy that encourages people to maintain a safety distance (often between one to two meters) from others. This policy differs by country, but

includes other policies that close cultural institutions (e.g., museums or libraries), or encourage establishments to reduce density, such as limiting restaurant hours.

- *no_gathering*: Represents a policy that prohibits any type of public or private gathering. (whether cultural, sporting, recreational, or religious). Depending on the country, the policy can prohibit a gathering above a certain size, in which case the number of people is specified by the *no_gathering_size* variable.
- *event_cancel*: Represents a policy that cancels a specific pre-scheduled large event (e.g., parade, sporting event, etc). This is different from prohibiting all events over a certain size.
- *religious_closure*: Represents a policy that prohibits gatherings at a place of worship, specifically targeting locations that are epicenters of COVID-19 outbreak. See the section on Korean policy for more information on this policy variable.
- *no_demonstration*: Represents a policy that prohibits protest-specific gatherings. See the section on Korean policy for more information on this policy variable.

A.3 Mobility data

Mobility data comes from three of the biggest internet companies - Google, Facebook and Baidu. These companies have millions of users accessing their social media, e-commerce and other digital platforms every day. These data are utilized to construct aggregated, anonymized user location and movement metrics for various geographic regions and countries. Descriptions follow, and Table S1 contains a summary of the data used for each country.

A.3.1 Google

Google mobility data summarizes time spent by their users each day after Feb 6, 2020 in various types of places, such as residential, workplaces and grocery stores.[32] Specifically, it provides the percentage change in number of visits and length of stay in each type of place, compared to a baseline value. The baseline is the value on the corresponding day of the week during the 5-week

period between Jan 3, 2020 and Feb 6, 2020. The metrics are available starting Feb 15, 2020 at the country (Administrative Level 0) and state level (Administrative Level 1) for over 135 countries. We also access county-level metrics (Administrative Level 2) for the US. Types of places include the following:

i. *Grocery & pharmacy*: Places like grocery markets, food warehouses, farmers markets, specialty food shops, drug stores, and pharmacies.

ii. *Parks*: Places like local parks, national parks, public beaches, marinas, dog parks, plazas, and public gardens.

iii. *Transit stations*: Places like public transport hubs such as subway, bus, and train stations.

iv. *Retail & recreation*: Places like restaurants, cafes, shopping centers, theme parks, museums, libraries, and movie theaters.

v. *Residential*: Places of residence.

vi. *Workplaces*: Places of work.

A.3.2 Facebook

Facebook summarizes and anonymizes its user data into useful metrics that can be used to evaluate the movement of people.[33] Our analysis uses data beginning March 5, Feb 23 and Feb 24, 2020 for France, Italy and South Korea respectively. Specifically, Facebook aggregates the number of trips between tiles of up to a resolution of 360 square meters. We aggregate these data to the level of administrative regions, constructing metrics for number of trips *between* as well as *within* these regions. We use the following variables from the data provided by Facebook:

i. *Date* - The day of the movement.

ii. *Starting Location* - The region or tile where the movement of the group started.

iii. *Ending Location* - The region or tile where the movement of the group ended.

iv. *Baseline Movement* - The total number of people who moved from Starting Location to Ending Location on average during the weeks before the disaster began.

v. *Crisis Movement* - The total number of people who moved from Starting Location to Ending Location during the time period specified

A.3.3 Baidu

Baidu provides aggregated user location data and mobility metrics via its Smart Eye Platform.[35] These data were scraped and publicly shared by the China Data Lab. The metrics represent movement in and out of major regions across China each day in terms of an aggregated mobility index.[44] Index values are available beginning Jan 1, 2020. Baidu does not disclose specific information regarding the construction of the index.

A.3.4 SafeGraph

SafeGraph data were generated by tracking anonymous mobile devices across US.[34] The mobility metrics are available starting January 1, 2020 for census block group. SafeGraph infers home location based on night time location of the device and uses that to impute average distance travelled per day by the devices in each census block. We aggregate this data to the state level (Administrative Level 1) for our analysis.

Table S1: **Mobility Data Sources -** Details of mobility data sources for each country. The table provides relevant dates and level of analysis used for the behavior model and infection model, respectively.

**Region**	**Mobility Data**	**Level of analysis**	**Type of mobility**	**Start Date**	**End Date**
**Behavior model**					
China	Baidu	ADM2	between	1/10/2020	3/6/2020
France	Facebook	ADM1	between	3/4/2020	4/8/2020
Italy	Facebook	ADM2	between	2/25/2020	4/8/2020
South Korea	Facebook	ADM2	between	2/23/2020	4/6/2020
United States	Google	ADM2	residential, retail, workplaces	3/3/2020	4/12/2020
World	Google	ADM0	residential, retail, workplaces	2/26/2020	3/28/2020
**Infection model**					
China	Baidu	ADM1, ADM2	between	2/12/2020	3/3/2020
France	Facebook	-	-	-	-
Italy	Facebook	ADM1, ADM2	between	3/31/2020	4/13/2020
South Korea	Facebook	-	-	-	-
United States	Google	ADM1, ADM2	-	3/17/2020	4/30/3020
World	Google	ADM0	-	mobility today >= 5%	5/29/2020 or until growth rates of new cases flatten

B Methods Summary

B.1 Behavior Model

The behavior model describes how human mobility changes as a result of NPIs ($\frac{\Delta behavior}{\Delta NPI}$ in equation (1)). The model is a commonly used reduced-form approach in econometrics. Details on the model and model estimation are presented below.

Model details:

1. The model used for each policy is $m_t = f(policy_t, X_t) + \epsilon_t$, where m_t is a measure of mobility behavior at time t, X_t represents control variables, and ϵ_t is the error. We use a linear functional form for $f(.)$ (see point 5 below).

2. The model is fit for each country at the sub-national level where granular policy and mobility data are available. For the rest of the world, use a panel regression model where the unit of observation is at the country by day level.

3. The *policy* variable is a vector with NPIs specific to each country, for each location and day. NPIs are continuous variables between 0 and 1 (inclusive) that indicate the intensity of the policy where 0 is no enforcement and 1 is fully enacted. In some instances, it may be desirable to gather multiple policies in a single variable (for example, business closure and restaurant closure) by taking the average, thus the maximum value of 1 would indicate that all policies are fully enacted.

4. The control variable X includes one-hot encodings of sub-national (or national) units and day-of-week variables. The former account for time-invariant factors (for example, socio-economic status, culture, public transportation availability) that impact mobility m, while the later control for weekly patterns in mobility (for example, less workplace related mobility on Sunday) that are common across location unit.

5. In summary, the model used is $m_{it} = \sum_{p=1}^{P_i} \beta_p policy_{p,it} + \gamma_i + \delta_t + \epsilon_{it}$, where i is the unit

of analysis, γ_i are unit-level fixed effects and δ_t are day-of-week fixed effects. P denotes the maximum number of policies ever enforced for a given region.

Steps for model estimation:

1. Estimate the average effect on mobility in all subsequent periods, $\hat{\beta_p}$, of each policy included in each model using the model described above, and ordinary least squares. The time period that we consider is the "first wave" of infections; specific dates are in Table S1.

2. Compute the combined effect of policies on human mobility by taking the sum across all $\hat{\beta_p}$ for a given region. Standard errors are computed using the variance covariance matrix of the coefficients.

B.2 Infection Model

Similar to the behavior model, the infection model is also a reduced-form approach, used to describe the relationship between infections and mobility behavior ($\frac{\Delta infections}{\Delta behavior}$ in equation (1)). Model details, as well as steps for model estimation, forecasting and cross-validation are outlined below. Also included are steps for data selection.

Model details:

1. The model used is $log(\frac{I_t}{I_{t-1}}) = g(mobility_t, X_t) + \epsilon_t$, where $log(\frac{I_t}{I_{t-1}})$ is the first-difference of log confirmed infections at time t, X_t represents control variables, and ϵ_t is the error. We use a linear functional form for $g(.)$ (see point 5 below).

2. The model is fit for each country at the sub-national level where granular infections and mobility data are available. For the global model, use a regression model where the unit of observation is at the country by day level.

3. The *mobility* variable is a vector with mobility rates specific to each country, for each location and day. Includes mobility measures averaged over lags 1-7, 8-14 and 15-21, respectively.

We use Google mobility data in its original form (percentage points), and take logs for the Facebook and Baidu mobility data.

4. The control variable X includes one-hot encodings of sub-national (or national) units, day-of-week variables, and indicators for changes in testing regimes.

5. In summary, the model used is $log(\frac{I_{it}}{I_{i,t-1}}) = \beta_1 m_{1-7,it} + \beta_2 m_{8-14,it} + \beta_3 m_{15-21,it} + \gamma_i + \delta_t + \phi_{it} + \epsilon_{it}$,
where i is the unit of analysis, γ_i are unit-level fixed effects, δ_t are day-of-week fixed effects, and $\phi_{it}$are indicators for changes in testing regimes.

The model is robust to systematic differences in infection tracking across locations, since the dependent variable is a growth rate. Regional fixed effects allow for location-specific underreporting; estimates are unbiased as long as the location-specific reporting rate remain constant over time. Major changes in testing regimes within a location are included in our model via unit-day specific dummy variables.

Steps for model estimation: The following steps are used to generate estimates of the average effect of each mobility variable on the growth rate of infections (see Figure S2). These are then used to estimate how a novel policy affecting mobility would alter future infections (Table 2).

1. Estimate the average effect of each mobility variable on the growth rate of infections, $\hat{\boldsymbol{\beta}} = \{\beta_1\ \beta_2\ \beta_3\}$, using the model described above, and ordinary least squares.

2. To estimate the potential effect of a mobility-reducing policy, use $\Delta I = h(\Delta m, \hat{\boldsymbol{\beta}})$, where ΔI is the change in number of infections, Δm is the anticipated change in mobility due to NPIs, and $\hat{\boldsymbol{\beta}}$ are estimated coefficients of the mobility variables. $h(.)$ is described in point 3 below.

3. Specifically, for Facebook and Baidu data, where mobility variables are in log form: at forecast day k, $\frac{I_{new}}{I_{original}} = e^{k\left(\sum_{l=1}^{3} \hat{\beta}_l \log(1+\Delta m_l)\right)}$, where Δm_l is the fractional change in the lth mobility variable (number of trips for all lags involved) (e.g., if the number of trips for all lags in the lth variable is reduced by 10%, $\Delta m_l = -.1$). For Google data: at forecast day k, $\frac{I_{new}}{I_{original}} = e^{k\left(\sum_{l=1}^{3} \hat{\beta}_l \Delta_l\right)}$, where Δm_l is the change in residential time over baseline for the lth

mobility variable (e.g., $\Delta m_l = .05$ means a 5% increase, say from 20% to 25% residential time over baseline, for all lags in the lth variable).

Steps for forecasting and cross-validation:

1. For a 20-day period (training data), fit a regression model as specified above, using ordinary least squares.

2. For a 10-day period (test data), multiply the coefficient estimates obtained from fitting the regression model on the training data with the observed predictor variables in the test data to obtain predictions of the infection rate (according to the equation in point 5 of *Model Details*).

3. For each test day, compute the percentage error compared to the ground-truth infection rate.

4. Perform cross-validation (i.e., robustness to train and test sample selection), by repeating the above for all 20-day training periods and 10-day forecast periods, limited by data availability.

5. Group percentage errors by day of forecast (from 1 to 10).

6. Repeat the above, using a baseline model which excludes mobility variables, i.e.,
   $log(\frac{I_{it}}{I_{i,t-1}}) = \gamma_i + \delta_t + \phi_{it} + \epsilon_{it}$.

In other words, the baseline model simply uses past infections to predict future infections. Smaller errors using the model including mobility variables would indicate that information on mobility improves forecasts. Examples of the results are in Figure 3, and forecasting errors are in 4.

Steps for data selection:

The time period that we consider (see Table S1) is the "first wave" of infections, and to demonstrate the utility of the mobility model, we focus on the period in which mobility starts falling as a result of lockdown measures imposed during this first wave, until right before mobility starts increasing again. This model can be refit to the local context of interest, using data that is representative of current conditions. For countries in which lockdown policy data are available, we include administrative regions after the lockdown policy has been implemented. For countries without policy data available on a granular enough level (US in this case), we use a start date of March 17 (the

results are robust to different start dates), or when Google residential mobility is at least 5 percent above baseline (world level). We select an end date that roughly corresponds to just before mobility picks back up. The reason for this choice is that in the phase in which mobility starts to increase, we might expect there to be other measures put in place, or other changes in behavior, such as contact tracing, mask wearing, and so forth, which justified the lifting of lockdown measures and subsequent increase in mobility. The relationship between mobility and cases might therefore be different than during the lockdown stage, suggesting that the model needs to be refit if we would like for it to be used during this period.

Now, we train each model using 20 days of training data, and forecast for up to 10 days into the future. To be included in the data used to train each model, we impose the following conditions at the level of the *administrative region.* For the administrative region to be included, for *all* 20 training days t,

1. $I_t \geq 10$
2. $I_{t-1} > 0$

   and for the world-level analysis only:

3. mobility$_t \geq 5$ percent, i.e., current day mobility is at least 5 percent above baseline.
4. $\left(\log \frac{I_t}{I_{t-1}}\right)_{i,1-14} \leq .03$ percent, i.e., the 14 days rolling average of the growth rate of cumulative active cases flattens.

These conditions also imply that I_t, I_{t-1} and the mobility variables have to be non-missing for all training days. These conditions have implications for predictions as well: if an administrative region is not included in the training data, predictions will not be generated for that region, because the region fixed effect would not be estimated for that region.

C Additional Figures

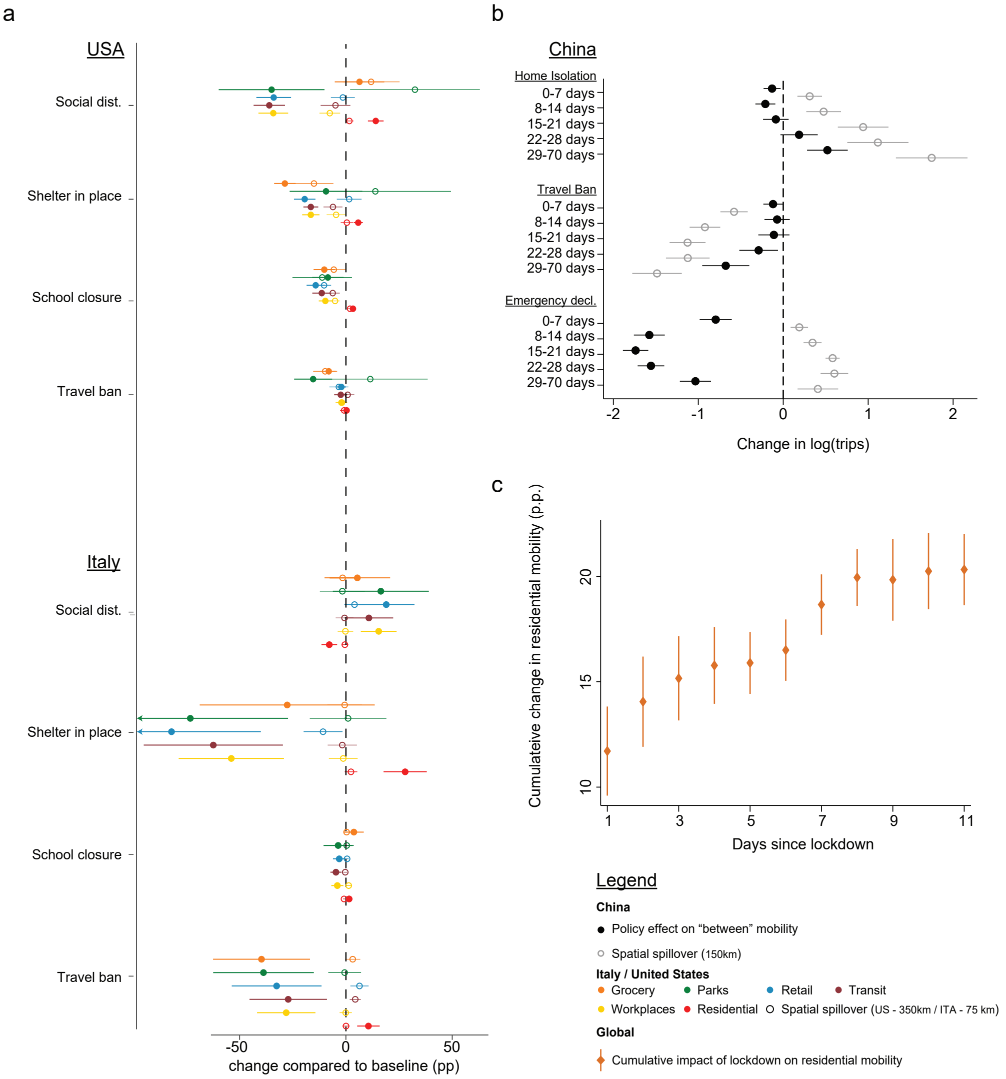

Figure S1: **Spatial and temporal spillover of policies**. (**a-b**) Solid markers indicate the direct impact of large policies on mobility. Hollow markers show the estimated effect of a policy on neighboring regions. Policies are jointly estimated at the local level for each country. In China (**b**), we also separately estimate the effect of each policy for each time period after the policy's implementation. (**c**) The impact of lockdown on the time spent at home is estimated using a country-level regression with 80 countries. We report the cumulative effect over time.

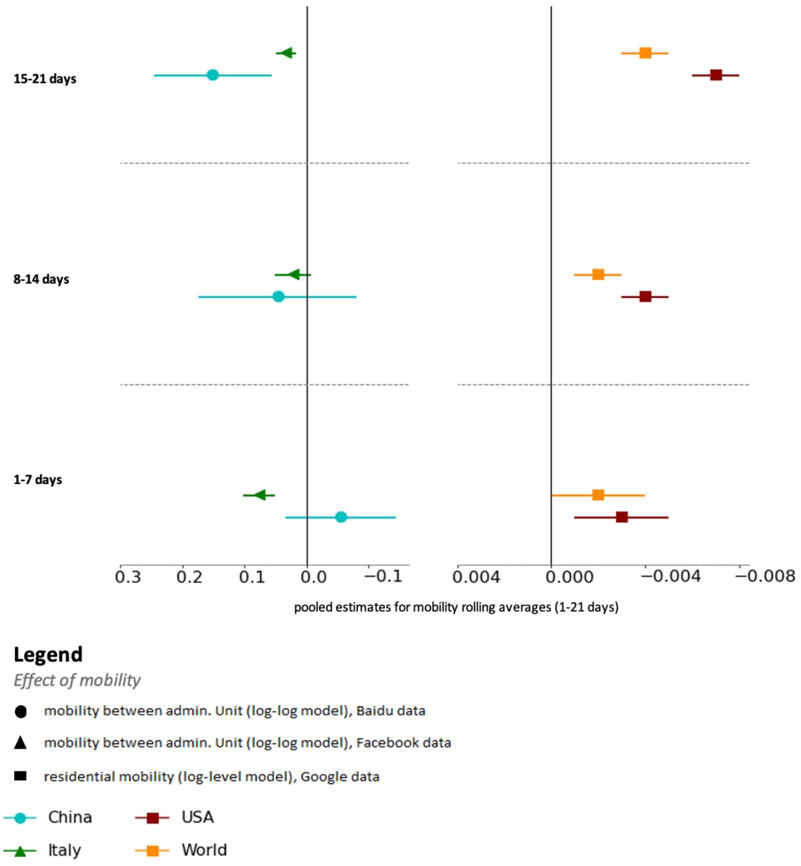

Figure S2: **Impact of mobility on the growth rate of COVID-19 cases**. Estimated impact of mobility on COVID-19 infection growth rate over time. Effects are estimated for each of the preceding three weeks (lags of 1 to 21 days), where the measure of mobility is either the number of trips between administrative units (left) or the amount of time spent at home (right). The impact of mobility is gradually increasing over time and is highest after 2 weeks.
